# Association between circulating fibroblast growth factor 21 and mortality in end-stage renal disease

**DOI:** 10.1371/journal.pone.0178971

**Published:** 2017-06-05

**Authors:** Marina Kohara, Takahiro Masuda, Kazuhiro Shiizaki, Tetsu Akimoto, Yuko Watanabe, Sumiko Honma, Chuji Sekiguchi, Yasuharu Miyazawa, Eiji Kusano, Yoshinobu Kanda, Yasushi Asano, Makoto Kuro-o, Daisuke Nagata

**Affiliations:** 1 Division of Nephrology, Department of Internal Medicine, Jichi Medical University, Shimotsuke, Tochigi, Japan; 2 Division of Anti-aging Medicine, Center for Molecular Medicine, Jichi Medical University, Shimotsuke, Tochigi, Japan; 3 Department of Nephrology, Japanese Red Cross Koga Hospital, Koga, Ibaraki, Japan; 4 Nasu-Minami Hospital, Nasukarasuyama, Tochigi, Japan; 5 JCHO Utsunomiya Hospital, Utsunomiya, Tochigi, Japan; 6 Division of Hematology, Department of Medicine, Jichi Medical University, Shimotsuke, Tochigi, Japan; 7 AMED-CREST, Japan Agency for Medical Research and Development, Chiyoda-ku, Tokyo, Japan; The University of Tokyo, JAPAN

## Abstract

Fibroblast growth factor 21 (FGF21) is an endocrine factor that regulates glucose and lipid metabolism. Circulating FGF21 predicts cardiovascular events and mortality in type 2 diabetes mellitus, including early-stage chronic kidney disease, but its impact on clinical outcomes in end-stage renal disease (ESRD) patients remains unclear. This study enrolled 90 ESRD patients receiving chronic hemodialysis who were categorized into low- and high-FGF21 groups by the median value. We investigated the association between circulating FGF21 levels and the cardiovascular event and mortality during a median follow-up period of 64 months. A Kaplan-Meier analysis showed that the mortality rate was significantly higher in the high-FGF21 group than in the low-FGF21 group (28.3% vs. 9.1%, log-rank, *P* = 0.034), while the rate of cardiovascular events did not significantly differ between the two groups (30.4% vs. 22.7%, log-rank, *P* = 0.312). In multivariable Cox models adjusted a high FGF21 level was an independent predictor of all-cause mortality (hazard ratio: 3.98; 95% confidence interval: 1.39–14.27, *P* = 0.009). Higher circulating FGF21 levels were associated with a high mortality rate, but not cardiovascular events in patient with ESRD, suggesting that circulating FGF21 levels serve as a predictive marker for mortality in these subjects.

## Introduction

Fibroblast growth factor 21 (FGF21), a member of the FGF family, is a hormone that regulates the glucose and lipid metabolism as well as the responses to several stresses [1–5]. FGF21 is secreted mainly by the liver and adipose tissue into the blood [3, 4, 6]. The cellular actions of FGF21 are mediated by FGF receptors (mainly FGFR1c) and βKlotho, a single-pass transmembrane protein that functions as an obligate co-receptor for FGF21 [1, 7].

Circulating FGF21 levels are elevated in various metabolic disease states, such as obesity, insulin resistance, and type 2 diabetes mellitus [8, 9]. In addition, the circulating FGF21 level increases progressively from the early to end stages of chronic kidney disease (CKD) and is associated with the renal function [10, 11]. Indeed, end-stage renal disease (ESRD) patients receiving dialysis showed 8- to 15-fold higher circulating FGF21 levels than normal subjects [9, 12], and circulating FGF-21 levels were inversely correlated with the residual renal function in peritoneal dialysis patients [13]. Furthermore, the FGF21 level is an independent biomarker of rapid CKD progression in patients at early stages of diabetic nephropathy [14, 15]. Recent studies showed that the circulating FGF21 level predicts the rates of cardiovascular events and mortality in patients with type 2 diabetes [16, 17] and coronary artery disease [18]. However, the impact of the circulating FGF21 level on the clinical outcome in patients with advanced CKD remains unclear.

In this study, we investigated the association of circulating FGF21 levels with the rates of cardiovascular events and mortality in ESRD patients receiving hemodialysis (HD).

## Materials and methods

### Study population

This retrospective cohort study included chronic 90 HD patients who were treated at Japanese Red Cross Koga Hospital (Ibaraki, Japan) and Nasu-Minami Hospital (Tochigi, Japan). The rationale, design, and data collection procedures of the study, which was performed at Japanese Red Cross Koga Hospital, have been described previously [19, 20]. The exclusion criteria for all participants were as follows: active malignancy, pulmonary disease, severe infectious disease, peritoneal dialysis, death within three months after study entry, and failure to cooperate with the study or provide consent to participate. From April 2004 to March 2005, 108 patients received dialysis at Japanese Red Cross Koga Hospital. Patients were excluded for the following reasons: hepatocellular carcinoma (n = 1), pulmonary hemorrhage (n = 1), fatal pneumonia (n = 1), peritoneal dialysis (n = 2), and death within 3 months (n = 2; sudden death [n = 1] and pneumonia [n = 1]). Thirty-eight patients were unable to give their consent or were unable to agree with the storage of their serum sample. From August to November 2014, 27 patients received HD at Nasu-Minami Hospital. All of these patients were enrolled in this study because the exclusion criteria were not applicable. Finally, a total of 90 patients (Japanese Red Cross Koga Hospital n = 63, Nasu-Minami Hospital n = 27) were included in this study.

The study was conducted in accordance with the Declaration of Helsinki. The research protocol was approved by the Medical Ethics Committees of Japanese Red Cross Koga Hospital and Nasu-Minami Hospital, and all of the patients provided their written informed consent.

### Data collection

Demographic and medical data, including age, gender, smoking history, and comorbid conditions, were obtained from the subjects' medical records and via standardized interviews. Body mass index (BMI) was calculated as the weight (kg) divided by the square of the height (m^2^). Blood samples were obtained before HD on the first dialysis day of the week. The blood samples that were used for plasma glucose measurement were collected in tubes containing sodium fluoride (a glycolysis inhibitor). These samples were separated from the cells within 30 min to prevent glycolysis [21]. Patients had been in the supine position for at least 10 minutes before blood collection. Aliquots of serum were obtained immediately at study entry and stored at −80°C until further use. The Kt/V urea index and normalized protein catabolism rate (nPCR), which integrate the efficiency of solute removal as urea clearance (K), treatment duration (T), and the patient's size as urea distribution volume (V), were determined using previously described formulas [22]. Blood pressure was measured before HD using a calibrated digital scale. The serum levels of FGF21 were determined using a sandwich enzyme-linked immunosorbent assay (ELISA) kit (Bio Vendor, Modrice, Czech Republic) in accordance with the manufacturer's instructions [9, 10, 23]. For long-term storage and measurement stability, only serum samples that have been stored at -70°C and which had never been thawed were used for the measurement. brain natriuretic peptide (BNP), Intact parathyroid hormone (PTH), insulin-like growth factor-1 (IGF-1) and serum insulin were measured using a commercial clinical diagnostic testing service (SRL, Inc., Tokyo, Japan). Diabetes mellitus was defined based on one of the following criteria: (i) a fasting plasma glucose level of ≥126 mg/dL; (ii) a 2-hour value of ≥200 mg/dL in a 75 g oral glucose tolerance test; or (iii) a casual plasma glucose level of ≥200 mg/dL [24]. The homeostasis model assessment of insulin resistance (HOMA-IR) value was calculated from the fasting concentrations of plasma glucose and serum insulin as follows: plasma glucose (mg/dL) × serum insulin (μIU/mL) / 405 [25].

### Outcome measurements

The primary outcomes were death and the first episode of nonfatal cardiovascular events, as described previously [19, 20]. Cardiovascular events were defined as angina or myocardial infarction; congestive heart failure requiring hospitalization; transient ischemic attacks; stroke; peripheral vascular disease, major arterial/venous thrombotic episode, and sudden death. For patients with multiple cardiovascular events, the time to the first episode was taken for survival analysis. Cardiac death was defined as death from any cardiovascular event except for non-heart based cardiovascular death such as stroke and peripheral vascular disease. Cardiovascular events and cause of death were assessed by the attending physicians who were unaware of serum FGF21. The information in the patients' medical charts was also included in the analysis. The follow-up was completed for all patients.

### Statistical analyses

The data are expressed as either the number of participants or the percentage of the study population. The remaining data are expressed as the mean ± standard deviation, or the median and interquartile IQR for variables with a non-normal distribution. One-way ANOVA was used to compare only 4 groups. The groups were compared using Student’s t-test for normal distributions and the Wilcoxon rank-sum test for non-normal distributions. The chi-square test was used to evaluate the proportional differences in categorical variables. The correlation between circulation FGF21 levels and associated clinical factors were analysed by the Pearson correlation and partial correlation. The subjects were categorized as follows: low-FGF21 group: subjects with a serum FGF21 level < 50th percentile; high-FGF21 group: subjects with a serum FGF21 level ≥ 50th percentile. We then assessed the impact of the serum FGF21 levels on the cumulative patient and cardiovascular event-free survival rates according to the Kaplan-Meier method combined with the log-rank test. Cox regression models were used to analyze the relationships between the all-cause mortality and the serum FGF21 levels. Because this study had only 17 death events, we were unable to enter more than 2 covariates into the multivariate analysis. Therefore, we adopted the prognostic models “J-DOPPS risk score” [26] and “ARO risk score” [27] in the Cox regression models as integrated confounding factors. The “J-DOPPS risk score” is a new risk model for predicting cardiovascular events among Japanese HD patients that consists of only six predictors: age, diabetes status, history of cardiovascular events, dialysis time per session, and serum phosphorus and albumin levels [26]. The cumulative risk points of this score range from 0 to 20 points, and it has dose-dependent association with cardiovascular events [26]. The “ARO risk score” is a mortality risk scores among European HD patients that includes well-known risk factors such as age, smoking status, BMI, history of cardiovascular events, vascular access, CRP, and serum albumin and creatinine levels [27]. Cumulative risk points (2-year risk points) range from -10 to 27 points, and patients with over 8 points have a high risk of all-cause mortality [27]. The raw points of these two prognostic models [26, 27] were used as a nominal variable in multiple Cox regression models. The results are presented as hazard ratio (HR) and 95% confidence interval (CI). The statistical analyses were performed using the JMP Pro 12.2.0 software program (SAS Institute, Cary, NC, USA). *P* values < 0.05 were considered to be statistically significant.

## Results

### Patient characteristics

**Fig 1:**
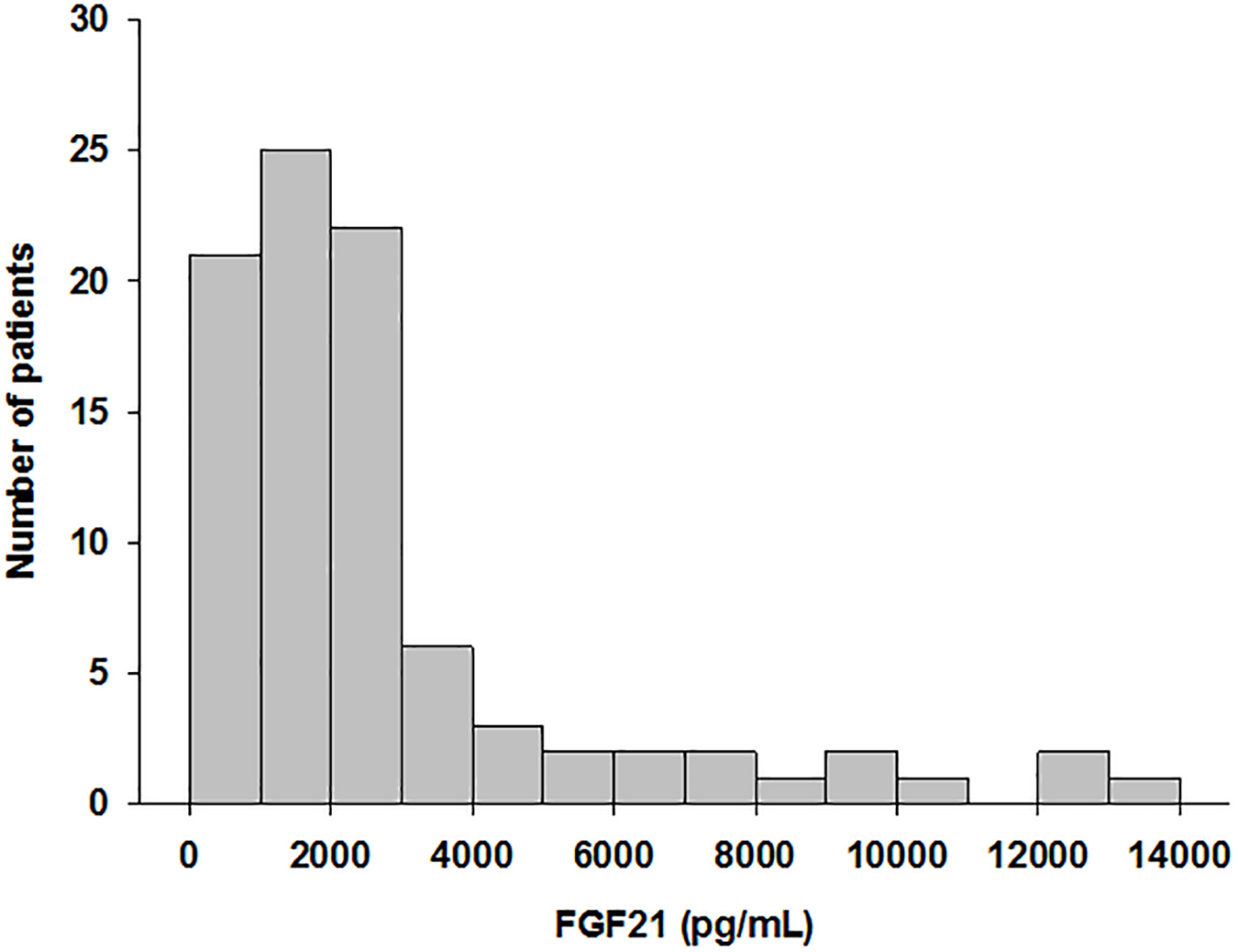
Distribution of circulating FGF21 levels in this study. FGF21 was detectable in the serum of all 90 patients, with serum concentrations ranging from 164 to 13214 pg/mL (median: 1981, interquartile range: 1034–2995 pg/mL).

A total of 90 HD patients were enrolled in this study. The mean age was 66.1 ± 12.9years (range 22–89 years), 58.9% were male, mean BMI was 21.7 ± 3.0, 42.2% was diabetes mellitus, duration of dialysis was 6.2 ± 5.7 years and 24.4% had a history of cardiovascular disease (Table 1). The underlying causes of CKD were diabetic nephropathy in 38 patients (42.2%), chronic glomerulonephritis in 36 patients (40.0%), hypertensive nephrosclerosis in 6 patients (6.7%), and other causes in 10 patients (11.1%). FGF21 was detectable in the serum of all 90 patients, with serum concentrations ranging from 164 to 13214 pg/mL (median: 1981, interquartile range [IQR]: 1034–2995 pg/mL) (Fig 1). Because the effective measurement range of the FGF21 assay was between 30 and 1920 pg/mL, serum samples with a high FGF21 level (>1920 pg/mL) were diluted appropriately with the assay buffer. All of the samples were over the detection limit, and 47 samples (52.2%) were over 1920 pg/mL. These samples were measured an additional two or three times after dilution and the average value was used. The serum FGF21 values for the different etiologies of ESRD not differ to a statistically significant extent: diabetic nephropathy, 2849 ± 3048 pg/mL; chronic glomeruronephritis, 2612 ± 2801 pg/mL; hypertensive nephrosclerosis, 1517 ± 977 pg/mL; and other renal disease, 3940 ± 3314 pg/mL (*P* = 0.415). The baseline characteristics of the participants are presented in Table 1, overall and by the median FGF21 levels. At baseline, uric acid and CRP were significantly higher, and serum insulin, HOMA-IR and the percentage of ARO risk score ≥ 8 were significantly lower in the high-FGF21 group than in the low-FGF21 group (Table 1). In contrast, no significant differences were observed in gender, BMI, duration of dialysis, serum albumin, intact PTH, IGF-1, total cholesterol, LDL-cholesterol, triglyceride levels or medications, including lipid-lowering therapy and cardiovascular medications (Table 1).

**Table 1:** Baseline characteristics of patients according to study group.

Variable	Total (n = 90)	Low-FGF21 (n = 44)	High-FGF21 (n = 46)	*P* value
Age (years)	66.1 ± 12.9	67.1 ± 13.3	65.6 ± 12.3	0.251
Male gender (%)	58.9	54.6	63.0	0.413
Dry weight (kg)	53.3 ± 11.2	53.0 ± 11.7	53.6 ± 10.9	0.800
BMI (kg/m^2^)	21.7 ± 3.0	21.6 ± 3.0	21.7 ± 3.1	0.447
Diabetes mellitus (%)	42.2	43.2	41.3	0.857
Duration of dialysis (years)	6.2 ± 5.7	6.3 ± 6.6	6.2 ± 4.7	0.442
History of cardiovascular disease (%)	24.4	22.7	26.1	0.711
Smoking (%)	64.8	56.8	72.7	0.117
Systolic blood pressure (mmHg)	152 ± 20	151 ± 19	152 ± 21	0.342
Diastolic blood pressure (mmHg)	79 ± 10	79 ± 11	79 ± 10	0.401
BNP (pg/mL)	193 (102–428)	175 (86–306)	218 (121–841)	0.081
Serum creatinine (mg/dL)	10.1 ± 2.8	9.6 ± 2.6	10.5 ± 2.9	0.067
Frequency of HD sessions (per week)	2.8 ± 0.4	2.8 ± 0.4	2.8 ± 0.4	0.426
Time of HD session (hour/session)	3.7 ± 0.4	3.7 ± 0.4	3.7 ± 0.4	0.300
Kt/V (per week)	1.36 (1.15–1.51)	1.37 (1.22–1.51)	1.31 (1.12–1.53)	0.457
nPCR (g/kg/day)	0.89 ± 0.21	0.89 ± 0.22	0.88 ± 0.19	0.318
Hemoglobin (g/dL)	9.7 ± 1.2	9.7 ± 1.1	9.8 ± 1.4	0.360
Serum albumin (g/dL)	3.8 ± 0.4	3.8 ± 0.5	3.7 ± 0.4	0.275
Uric acid (mg/dL)	6.8 ± 1.6	6.4 ± 1.3	7.1 ± 1.8	0.037
Serum calcium (mg/dL)	8.8 ± 1.1	8.8 ± 1.2	8.8 ± 1.1	0.413
Serum phosphorus (mg/dL)	4.8 ± 1.2	4.9 ± 1.3	4.7 ± 1.1	0.252
Intact PTH (pg/mL)	111 (57–197)	113 (43–226)	100 (74–197)	0.660
IGF-1 (ng/mL)	106 (76–166)	108 (81–168)	104 (74–159)	0.351
CRP (mg/dL)	0.07 (0.03–0.16)	0.05 (0.03–0.10)	0.09 (0.04–0.30)	0.031
Glucose (mg/dL)	124 (104–156)	124 (108–155)	122 (103–156)	0.948
Serum insulin (mIU/mL)	28.5 (15.9–44.8)	34.1 (21.7–50.2)	23.7 (10.3–37.0)	0.007
HOMA-IR	8.4 (5.3–14.6)	9.7 (6.0–17.5)	7.1 (3.8–11.6)	0.014
Total cholesterol (mg/dL)	150 (134–174)	144 (135–177)	151 (128–171)	0.865
HDL-cholesterol (mg/dL)	38 (31–46)	37 (30–48)	38 (31–45)	0.929
LDL-cholesterol (mg/dL)	77 (66–91)	77 (67–89)	77 (61–92)	0.625
Triglyceride (mg/dL)	74 (50–110)	74 (53–104)	76 (45–114)	0.774
FGF21 (pg/mL)	1981 (1034–2995)	1029 (704–1518)	2989 (2184–5973)	<0.001
J-DOPPS risk score (points)	5.9 ± 3.1	5.9 ± 3.0	5.9 ± 3.2	0.941
ARO risk score (points)	4.4 ± 3.5	4.7 ± 3.8	4.1 ± 3.2	0.213
ARO risk score ≥ 8 (%)	18.9	27.3	10.9	0.045
Medications				
Statin (%)	11.1	13.6	8.7	0.455
ARBs/ACEIs (%)	51.1	47.7	54.4	0.530
β-blocker (%)	10.0	4.6	15.2	0.083
Anti-platelet drugs (%)	27.1	33.3	20.9	0.197
Vitamin D analogues (%)	48.9	52.3	45.7	0.530
Phosphate binders (%)	81.1	77.3	84.8	0.363

Variables are presented as mean ± standard deviation, or as median (interquartile range), as appropriate. BMI: body mass index; BNP: brain natriuretic peptide; HD: hemodialysis; nPCR: normalized protein catabolism rate; PTH: parathyroid hormone; IGF-1: insulin-like growth factor-1; ARBs: angiotensin receptor blockers; ACEIs: angiotensin converting enzyme inhibitors

At baseline, circulating FGF21 levels correlated positively with BNP, uric acid and CRP (Table 2). The correlation between circulating FGF21 levels and these variables remained unchanged after adjustment for age, gender and BMI (Table 2).

**Table 2:** Correlations coefficients of circulating FGF21 levels for associated clinical factors.

Variable	No Adjustment^a^		Adjusted by age, gender, BMI^b^	
	R	*P* value	R	*P* value
Age (years)	-0.035	0.743		
Male gender	-0.032	0.745		
BMI (kg/m^2^)	0.011	0.919 0.27		
Systolic blood pressure (mmHg)	0.121	0.257		
Log BNP	0.346	<0.001	0.388	<0.001
Hemoglobin (g/dL)	-0.098	0.356 0		
Serum albumin (g/dL)	-0.094	0.376		
Uric acid (mg/dL)	0.210	0.047	0.225	0.047
Log CRP (mg/dL)	0.330	0.002	0.349	0.002
Triglyceride (mg/dL)	-0.017	0.873		
HDL-cholesterol (mg/dL)	-0.111	0.298		
LDL-cholesterol (mg/dL)	0.031	0.774		

Log transformed FGF21 levels were used for the analysis.

^a^Pearson correlation analysis was used.

^b^Partial correlation was used.

BMI: body mass index; BNP: brain natriuretic peptide

### Mortality and cardiovascular events

**Fig 2:**
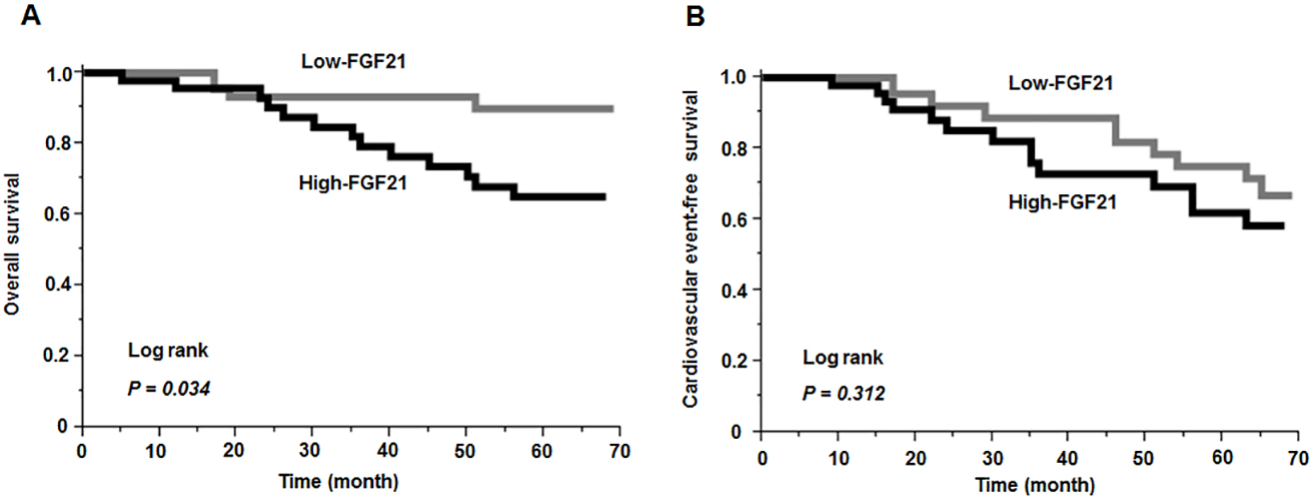
Association between circulating FGF21 and outcomes. The Kaplan-Meier analysis showed that the mortality rate was significantly higher in the high-FGF21 group than in the low-FGF21 group (log-rank, *P* = 0.034) (A), while the cardiovascular event-free survival rate did not significantly differ between the two groups (log-rank, *P* = 0.312) (B).

Seventeen patients (18.9%) died during the median follow-up period of 64 months (IQR: 21–66): 4 patients in the low-FGF21 group and 13 patients in the high-FGF21 group. Three patients (two congestive heart failure, one sudden death due to hyperkalemia) in the low-FGF21 group and eight patients (four heart failure, one stroke, one peripheral vascular disease, one artery/venous thrombosis, one sudden death due to rupture of aneurysm) in the high-FGF21 died of cardiovascular events, while one patient (pneumonia) in the low-FGF21 group and five patients (three cancers, two sepsis) in the high-FGF21 group died of non-cardiovascular events. It is noteworthy that all of the cancer deaths occurred in the high-FGF21 group. Additionally, in the high-FGF21 group, the percentage of non-cardiovascular deaths among total deaths (38.5%) was numerically higher in comparison to the low-FGF21 group (25.0%). Ten patients (congestive heart failure, n = 5; angina, n = 2; stroke n = 1; transient ischemic attack, n = 1; sudden death, n = 1) in the low-FGF21 group and 14 patients (congestive heart failure, n = 9; myocardial infarction, n = 2; stroke, n = 1; arterial/venous thrombotic episode, n = 1; one sudden death, n = 1) in the high-FGF21 group developed cardiovascular events. A Kaplan-Meier analysis showed that the mortality rate was significantly higher in the high-FGF21 group than in the low-FGF21 group (28.3% [n = 13] vs. 9.1% [n = 4], log-rank, *P* = 0.034) (Fig 2A), while the rate of cardiovascular events did not significantly differ between the two groups (30.4% [n = 14] vs. 22.7% [n = 10], log-rank, *P* = 0.312) (Fig 2B). Furthermore, the cardiac death rate was similar between the two groups (6.8% [n = 3] vs. 8.7% [n = 4], log-rank, *P* = 0.715). A univariate Cox regression analysis showed that age, diabetes mellitus, history of cardiovascular disease, low IGF-1, high CRP, high glucose, low LDL-cholesterol, J-DOPPS risk score, ARO risk score and high FGF21 were predictive for the all-cause mortality (Table 3). High FGF21 levels were significantly associated with the all-cause mortality (HR 3.16; 95% CI 1.12–11.22, *P* = 0.029) compared with low FGF21 levels (Table 4). After adjusting for the J-DOPPS risk score, a high FGF21 level was an independent predictor of the all-cause mortality (HR: 3.28; 95% CI: 1.14–11.54, *P* = 0.026) (Table 4). In addition, after adjustment for the ARO risk score, a high FGF21 level was an independent predictor of the all-cause mortality (HR: 3.98; 95% CI: 1.39–14.27, *P* = 0.009) (Table 4).

**Table 3:** Univariate Cox regression analyses of all-cause mortality.

Variable	All-cause mortality	
	HR (95% CI)	*P* value
Age (per year)	1.08 (1.03–1.15)	0.002
Male gender (vs female)	2.19 (0.81–6.91)	0.124
BMI (per kg/m^2^)	1.00 (0.85–1.15)	0.993
Diabetes mellitus	2.70 (1.03–7.46)	0.043
History of cardiovascular disease	3.02 (1.03–8.08)	0.045
Smoking	1.05 (0.40–3.04)	0.929
Systolic blood pressure (per 10 mmHg)	0.95 (0.74–1.20)	0.655
BNP (per 10 pg/mL)	1.00 (0.999–1.004)	0.138
Serum creatinine (mg/dL)	0.86 (0.72–1.02)	0.094
Kt/V (per week)	1.08 (0.19–6.78)	0.928
nPCR (per g/kg/day)	0.37 (0.03–5.43)	0.469
Hemoglobin (per g/dL)	0.89 (0.62–1.28)	0.523
Serum albumin (per g/dL)	0.81 (0.23–2.69)	0.742
Uric acid (per mg/dL)	1.01 (0.73–1.36)	0.964
IGF-1 (per 10 ng/mL)	0.86 (0.75–0.95)	0.002
CRP (per 0.1 mg/dL)	1.19 (1.05–1.32)	0.008
Glucose (per 1 mg/dL)	1.01 (1.001–1.017)	0.038
Serum insulin (per 1 mIU/mL)	0.988 (0.964–1.008)	0.266
HOMA-IR	0.99 (0.93–1.05)	0.809
Triglyceride (per 10 mg/dL)	0.96 (0.85–1.05)	0.446
HDL-cholesterol (per 10 mg/dL)	0.88 (0.57–1.29)	0.521
LDL-cholesterol (per 10 mg/dL)	0.75 (0.56–0.97)	0.026
Log FGF21	3.63 (1.04–12.74)	0.043
J-DOPPS risk score (per 1 point)	1.27 (1.09–1.49)	0.002
ARO risk score (per 1 point)	1.38 (1.15–1.71)	0.0003
Statin (No)	1.94 (0.39–34.92)	0.522
ARBs/ACEIs (No)	1.91 (0.73–5.55) 55)	0.202
β-blocker (No)	0.63 (0.18–3.97)	0.557
Anti-platelet drugs (No)	0.43 (0.16–1.29)	0.127

HR, hazard ratio; 95% CI, 95% confidence interval

BMI: body mass index; BNP: brain natriuretic peptide; nPCR: normalized protein catabolism rate; IGF-1: insulin-like growth factor-1; ARBs: angiotensin receptor blockers; ACEIs: angiotensin converting enzyme inhibitors

**Table 4:** Multivariate Cox regression analyses of all-cause mortality.

Models	Low-FGF21	High-FGF21	
		HR (95% CI)	*P* value
Crude	Referent	3.16 (1.12–11.22)	0.029
Crude + J-DOPPS risk score^a^	Referent	3.28 (1.14–11.54)	0.026
Crude + ARO risk score^b^	Referent	3.98 (1.39–14.27)	0.009

HR, hazard ratio; 95% CI, 95% confidence interval

^a^The J-DOPPS risk score consists of age, diabetes status, history of cardiovascular events, dialysis time per session, and serum phosphorus and albumin levels.

^b^The ARO risk score consists of age, smoking status, BMI, history of cardiovascular events and cancer, CKD etiology, vascular access, actual blood flow, hemoglobin, ferritin, CRP, and serum albumin and creatinine levels.

## Discussion

This study shows that circulating FGF21 levels predict the mortality rate in ESRD. Recent studies have reported that an elevated circulating FGF21 level predicts cardiovascular events and death in patients with type 2 diabetes and coronary artery disease, including early-stage CKD patients [16–18]. However, the link between FGF21 and the rate of cardiovascular events or mortality in patients with advanced CKD has not yet been evaluated. Therefore, to our knowledge, this is the first report evaluating the clinical impact of elevated circulating FGF21 levels on the long-term outcomes in ESRD.

FGF21 is mainly secreted by the liver in response to injury, systemic inflammation like sepsis, cancer, and kidney dysfunction [5, 23, 28–30]. An increase in the FGF21 level might be viewed as part of the defense mechanism for protecting tissues from damage. Johnson et al. reported that an increase in the pancreatic FGF21 expression was observed in mice with acute pancreatitis, which protected the pancreas from further damage [31]. Ye et al. showed that liver damage induced by acetaminophen in FGF21 knockout mice was more prominent than in wild-type mice, indicating that the induction of hepatic FGF21 expression was protective [32]. However, among critically ill patients, the circulating FGF21 levels in non-surviving patients (i.e. the sickest patients) were higher than in the surviving patients, suggesting that FGF21 can be regarded as a stress- or cell damage-induced hormone, but FGF21 does not act as a survival factor in a critically ill setting [28, 33]. Similarly, our data showed that an increase in the circulating FGF21 levels predicts a high mortality in ESRD, which indicates that patients with higher circulating FGF21 levels suffer from more severe stress and therefore fail to survive. In addition, interestingly, the percentage of patients with over 8 points of ARO risk score [27], which certainly predicts all-cause mortality in this population, was significantly lower in the high-FGF21 group than in the low-FGF21 group. Nevertheless, higher circulating FGF21 levels were associated with a high mortality rate, suggesting the strong effect of FGF21 on mortality independently of well-known risk factors.

The present study did not show any association between elevated circulating FGF21 levels and adverse cardiovascular events. Previous *in vivo* and *in vitro* studies have shown that FGF21 is protective for cardiomyocytes. A recent notable study showed that mice lacking FGF21 had enhanced cardiac hypertrophy; however, treatment with FGF21 in mice protected against this hypertrophic response, suggesting the cardioprotective role of FGF21 [6]. That study also showed that FGF21 was expressed and secreted by cardiac cells in response to cardiac hypertrophy, myocardial ischemia, and infarction [6]. Another study in mice showed that the expression of FGF21 in the liver and the serum levels of FGF21 were increased in response to experimental myocardial ischemia, and the administration of FGF21 was shown to mitigate myocardial infarction [34]. In the study, the suppression of FGF21 expression by small interfering (si)RNA-mediated gene silencing resulted in a significant increase in the fraction of myocardial infarcts [34]. The activation of the mitogen-activated protein kinase signaling via the activation of FGFR1c/*β*Klotho for preventing cardiac hypertrophy [6], the Sirt1-PPARα pathway in the regulation of FGF21 expression in the heart [6], and the FGFR1/*β*Klotho–PI3K-Akt1-BAD signaling network in myocardial ischemia/reperfusion injury [35, 36] are pivotal pathways that protect cardiac cells from damage. These *in vivo* and *in vitro* studies suggest that FGF21 may have some beneficial effect on the heart in human studies. In addition, the circulating FGF21 levels at baseline in our study were positively associated with the log BNP after adjustment for age, gender and BMI, suggesting a relationship between FGF21 and the cardiac function or impairment. However, we detected no apparent cardioprotective properties of FGF21 during the follow-up period. Therefore, the role of FGF21 as an organ protector in ESRD remains uncertain. Further studies in large populations or administration of recombinant FGF21 to ESRD patients will be needed to evaluate the cardioprotective properties of FGF21.

Several clinical studies have shown the association between circulating FGF21 and CKD. A cross-sectional study in community-dwelling adults reported that serum FGF21 is independently associated with the renal function and CKD [11]. In a study with CKD patients and healthy controls, plasma FGF21 levels were significantly increased with the development of early- to end-stage CKD and were independently associated with the renal function [10]. In ESRD patients receiving peritoneal dialysis, the circulating FGF-21 levels were also inversely correlated with the residual renal function [13]. The present study also showed that plasma FGF21 levels in end-stage CKD patients are about 10-fold higher than those in normal subjects and about 4.5-fold higher than those in early-stage CKD patients [10]. Two other studies in ESRD patients on dialysis showed that the FGF21 levels were about 15-fold higher in chronic HD patients and about 8-fold higher in peritoneal dialysis patients than in normal subjects [12, 37]. Furthermore, prospective cohort studies in type 2 diabetes have shown that the circulating FGF21 level is a marker of rapid CKD progression, remaining significant even after adjustment for the baseline glomerular filtration rate [14, 15]. This result suggests that elevated FGF21 levels might reflect the processes that are causally related to renal dysfunction in type 2 diabetes patients, although reduced renal clearance is suggested as a major cause of FGF21 elevation [10, 23]. In addition, FGF21 elevation may be linked to certain ESRD-related factors, such as uremic toxin, more extensive inflammation, and renal anemia, although the correlation between FGF21 and these factors has not yet been evaluated. Similar to previous reports of FGF21 in ESRD [9, 12], the circulating FGF21 concentration in our study was markedly high (median: 1981 pg/ml). The residual renal function may be similar between the high- and low-FGF21 groups, as not only serum creatinine but also markers of the nutritional status and dialysis efficacy such as body weight (dry weight), BMI, Kt/V, nPCR and serum albumin were all similar between the two groups. Therefore, the residual renal function may be similar between the two groups, suggesting that renal excretion of FGF21 is not a major determinant of the circulating levels in ESRD patients. Recent findings suggest that the FGF21 is increased in four main circumstances: 1) mitochondrial diseases; 2) oxidative stress; 3) physically stressful situations, such as ketogenic diets and lactation; and 4) pathological physically stressful situations, such as obesity and critical illness [38]. Among these circumstances, oxidative stress may be particularly important as a regulator in ESRD patients, as the patients are subjected to enhanced oxidative stress as a result of reduced anti-oxidant systems and increased pro-oxidant activity [39]. Furthermore, the severity of oxidative stress and chronic inflammation may determine the circulating FGF21 levels in ESRD patients. Indeed, our preliminary data showed that sleep-disordered breathing, an important mediator for accelerating oxidative stress and chronic inflammation [40], is associated with circulating FGF21 levels. Therefore, interventional treatment of sleep-disordered breathing, such as continuous positive airway pressure, may be a promising way of reducing circulating FGF21 levels.

Previous studies have shown the circulating FGF21 levels to be elevated in impaired glucose tolerance and diabetes [8, 9, 41]. However, in our study, the HOMA-IR level was lower in the high-FGF21 group, and the number of patients with diabetes was closely comparable between the high- and low-FGF21 groups. The key factors explaining these differences are considered to be the presence of “obesity” and “ESRD”. A community-based study (BMI > 25 kg/m^2^ 54.8%) showed a positive correlation between the serum FGF21 and HOMA-IR levels, but this correlation became insignificant after adjusting for the BMI [8]. This means that obesity itself may regulate not only insulin sensitivity, but also the serum FGF21 levels. On the other hand, our study population mainly consisted of non-obese ESRD patients (average BMI 21.7±3.0 kg/m^2^). The circulating FGF21 levels in ESRD tend to be dramatically elevated [12, 37], and certain ESRD-related factors, such as uremic toxin, more extensive inflammation, and renal anemia, may affect such an elevation. Because FGF21 stimulates the glucose uptake into adipose tissue in a dose-dependent manner [2], the elevated circulating FGF21 levels in the high-FGF21 group might have ameliorated insulin sensitivity. This mechanism might lead to a similar number of diabetes patients between the high- and low-FGF21 groups, although patients with higher FGF21 levels might have been more stress conditions.

Several limitations associated with the present study warrant mention. First, this study had a relatively small sample size and a small number of events. Therefore, further studies with a larger sample size are needed to confirm the association between the circulating FGF21 levels and mortality and cardiovascular events in ESRD. In particular, the statistical significance for cardiovascular events may change in larger studies, as the cardioprotective properties of FGF21 have been reported in recent *in vivo* and *in vitro* studies [6, 34–36]. Second, we cannot determine whether or not circulating FGF21 played a causal role in the outcome, because this was a cohort study. Further interventional studies involving, for example, the administration of recombinant FGF21 [42] and renal transplantation to reduce the circulating FGF21 levels are necessary to evaluate the therapeutic benefits of FGF21 on the cardiovascular event and mortality rates in ESRD. Third, details of the baseline cardiac function using echocardiography were lacking. Because we focused on the cardiovascular outcome, details of the cardiac function in addition to the BNP levels are necessary. Fourth, some blood samples might have been non-fasting samples that were collected just before HD with a regular meal pattern. Non-fasting samples might have affected the blood glucose, HOMA-IR and serum FGF21 levels [43]. On the other hand, it has been reported that the blood glucose level significantly decreases during a HD session that causes hypoglycemia [44]. Thus, we just used pre-dialysis blood samples, which might have included non-fasting samples. However, the timing of sample collection might have affected our results and should be recognized as a study limitation. Fifth, the setting of the exclusion criteria might have affected the results of all-cause mortality, because patients with active malignancy, pulmonary disease, or severe infectious disease and those who died within three months after entry into the study were excluded from the study. Although we focused on the effects of circulating FGF21 on the long-term outcomes in HD patients without life-threatening diseases, it will be necessary to include such patients in future studies. Sixth, the two prognostic models “J-DOPPS risk score” [26] and “ARO risk score” [27] used in the Cox regression models as integrated confounding factors do not include potential confounders such as sex, blood pressure and LDL cholesterol. This is a limitation of the statistical analysis. Seventh, the circulating FGF21 levels were determined in part using long-term storage samples. Although these serum samples are stored at -70°C and had never been thawed, in accordance with the manufacture’s instructions, the sample stability may not be completely ensured.

## Conclusions

We conclude that higher circulating FGF21 levels were associated with a higher mortality rate but not with a higher cardiovascular event rate in patients with ESRD. This study suggests that circulating FGF21 levels may be a predictive marker of the clinical outcomes. Further studies as interventional trials, involving for example the administration of FGF21 and renal transplantation, are required to evaluate the detailed clinical significance of FGF21.
